# Supplementary material for: Linear mapping approximation of gene regulatory networks with stochastic dynamics
Source: Nat Commun. 2018 Aug 17;9:3305. doi: 10.1038/s41467-018-05822-0 (PMC6098115; doi:10.1038/s41467-018-05822-0)
Supplement: Supplementary file 1 — Supplementary Information [file 41467_2018_5822_MOESM1_ESM.pdf]

Supplementary Information:
Linear mapping approximation of gene regulatory networks with stochastic dynamics

Cao *et. al.*

Supplementary Note 1 Exact solution for linear GRN with protein bursts

Here we present the exact time-dependent solution to the CME of the linear GRN resulting from the LMA mapping of the nonlinear feedback loop (see Eq. 20 in the main text).

By defining the generating functions $G_0(z,t) = \sum_{n_\mathrm{p}=0}^{\infty} z^{n_\mathrm{p}} P_0(n_\mathrm{p}, t)$ and $G_1(z,t) = \sum_{n_\mathrm{p}=0}^{\infty} z^{n_\mathrm{p}} P_1(n_\mathrm{p}, t)$, we can convert the master equations (Eqs. (21-22) in the main text) into the following first-order partial differential equations:

$$\begin{cases} \partial_t G_0 = \rho_\mathrm{u}\tilde{\psi}G_0 - \rho_\mathrm{u}G_0 - w\partial_w G_0 - \bar{\sigma}_\mathrm{b}G_0 + \sigma_\mathrm{u}G_1, \\ \partial_t G_1 = \rho_\mathrm{b}\tilde{\psi}G_1 - \rho_\mathrm{b}G_1 - w\partial_w G_1 + \bar{\sigma}_\mathrm{b}G_0 - \sigma_\mathrm{u}G_1, \end{cases} \tag{1}$$

where $\tilde{\psi}(w) = (1-bw)^{-1}$, $w = z-1$ and b is the mean size of bursts. Let $G_0 = (1-bw)^{-\rho_\mathrm{b}}\tilde{G}_0$ and $G_1 = (1-bw)^{-\rho_\mathrm{b}}\tilde{G}_1$, then Eq. (1) is equivalent to:

$$\begin{cases} \partial_t \tilde{G}_0 = -\rho_\Delta b\tilde{\psi}w\tilde{G}_0 - w\partial_w\tilde{G}_0 - \bar{\sigma}_\mathrm{b}\tilde{G}_0 + \sigma_\mathrm{u}\tilde{G}_1, \\ \partial_t \tilde{G}_1 = -w\partial_w\tilde{G}_1 + \bar{\sigma}_\mathrm{b}\tilde{G}_0 - \sigma_\mathrm{u}\tilde{G}_1. \end{cases} \tag{2}$$

Summing up the equations in (2):

$$\partial_t\tilde{G} + w\partial_w\tilde{G} = -\rho_\Delta b\tilde{\psi}w\tilde{G}_0, \tag{3}$$

where $\tilde{G} = \tilde{G}_0 + \tilde{G}_1$. Replacing $\tilde{G}_0$ in the first equation of (2) with $\tilde{G}$ by means of Eq. (3), then we have

$$\begin{aligned} &\partial_t\tilde{G}_0 + w\partial_w\tilde{G}_0 + \rho_\Delta b\tilde{\psi}w\tilde{G}_0 + (\bar{\sigma}_\mathrm{b} + \sigma_\mathrm{u})\tilde{G}_0 - \sigma_\mathrm{u}\tilde{G} \\ =&\partial_t\tilde{G}_0 + w\partial_w\tilde{G}_0 + (\bar{\sigma}_\mathrm{b} + \sigma_\mathrm{u})\tilde{G}_0 - \sigma_\mathrm{u}\tilde{G} - \partial_t\tilde{G} - w\partial_w\tilde{G} \\ =&-(\rho_\Delta b\tilde{\psi}w)^{-1}(\partial^2_{tt}\tilde{G} + w\partial^2_{wt}\tilde{G}) \\ &- w\partial_w[(\rho_\Delta b\tilde{\psi}w)^{-1}](\partial_t\tilde{G} + w\partial_w\tilde{G}) - (\rho_\Delta b\tilde{\psi})^{-1}(\partial^2_{wt}\tilde{G} + \partial_w\tilde{G} + w\partial^2_{ww}\tilde{G}) \\ &- (\bar{\sigma}_\mathrm{b} + \sigma_\mathrm{u})(\rho_\Delta b\tilde{\psi}w)^{-1}(\partial_t\tilde{G} + w\partial_w\tilde{G}) \\ &- \sigma_\mathrm{u}\tilde{G} - \partial_t\tilde{G} - w\partial_w\tilde{G} \\ =&0. \end{aligned}$$

Simplifying the above equation, we obtain a single parabolic partial differential equation in $\tilde{G}$:

$$(1-bw)\partial^2_{tt}\tilde{G} + 2(1-bw)w\partial^2_{wt}\tilde{G} + (1-bw)w^2\partial^2_{ww}\tilde{G} - q\partial_t\tilde{G} - (q-(1-bw))w\partial_w\tilde{G} + \rho_\Delta\sigma_\mathrm{u}bw\tilde{G} = 0, \tag{4}$$

where q is defined as $q = 1 - (\sigma_\mathrm{u} + \bar{\sigma}_\mathrm{b})(1-bw) - \rho_\Delta bw$. Subsequently, we apply the transformation that $\tilde{G}(w,t) = \tilde{G}(u,v)$ with $u = \ln w - t$ and $v = 1 - bw$, and it immediately follows that

$$v(1-v)\partial^2_{vv}\tilde{G} + [1 - \rho_\Delta - (\bar{\sigma}_\mathrm{b} + \sigma_\mathrm{u} - \rho_\Delta + 1)v]\partial_v\tilde{G} + \rho_\Delta\sigma_\mathrm{u}\tilde{G} = 0,$$

whose general solution can be represented as

$$\tilde{G} = f(u)y_1 + g(u)y_2,$$

where y_1 and y_2 have the form:

$$\begin{cases} y_1 = {}_2F_1(\alpha, \beta; \bar{\sigma}_\mathrm{b} + \sigma_\mathrm{u}; bw), \\ y_2 = (bw)^{1-\sigma_\mathrm{u}-\bar{\sigma}_\mathrm{b}}{}_2F_1(1-\rho_\Delta-\alpha, 1-\rho_\Delta-\beta; 2-\sigma_\mathrm{u}-\bar{\sigma}_\mathrm{b}; bw). \end{cases}$$

Note that ${}_2F_1$ is the hypergeometric function, and the parameters α and β should be solved from the

simultaneous set of equations:

$$\begin{cases} \alpha+\beta =\sigma_{\rm u}+\bar{\sigma}_{\rm b}-\rho_\Delta, \\ \alpha\beta =-\rho_\Delta\sigma_{\rm u}. \end{cases}$$

The functions f and g are to be determined according to the given initial conditions.

Plugging the solution for $\tilde{G}$ in Eq. (3), we find a solution for G_0 of the form:

$$G_0=-(1-bw)^{-\rho_{\rm b}}(\rho_\Delta b\tilde{\psi})^{-1}[f(u)\partial_w y_1+g(u)\partial_w y_2],$$

where we used the fact that

$$\begin{aligned} &\partial_t\tilde{G}+w\partial_w\tilde{G} \\ =&-\dot{f}y_1-\dot{g}y_2+w[w^{-1}\dot{f}y_1+w^{-1}\dot{g}y_2]+wf(u)\partial_w y_1+wg(u)\partial_w y_2 \\ =&wf(u)\partial_w y_1+wg(u)\partial_w y_2, \end{aligned}$$

where $\dot{f},\dot{g}$ are the derivatives of f and g with respect to u, respectively.

Suppose that the initial condition is $G_0(w,0)=1$ and $G(w,0)=1$, which means initially we have zero protein and the promoter is in state G. Note that at $t=0$, $u=\ln w$; hence, $f(u)$ and $g(u)$ are equivalent to $f(w)$ and $g(w)$. Plugging these initial conditions in the equations for G and G_0 obtained above, it follows that $f(w)$ and $g(w)$ can be solved from the following linear equations:

$$\begin{cases} y_1 f(w)+y_2 g(w)=(1-bw)^{\rho_{\rm b}}, \\ \partial_w y_1 f(w)+\partial_w y_2 g(w)=-(\rho_\Delta b\tilde{\psi})(1-bw)^{\rho_{\rm b}}. \end{cases}$$

The solution is:

$$\begin{cases} f(w)=\dfrac{[(\rho_\Delta b\tilde{\psi})y_2+\partial_w y_2](1-bw)^{\rho_{\rm b}}}{y_1\partial_w y_2-y_2\partial_w y_1}, \\ g(w)=-\dfrac{[(\rho_\Delta b\tilde{\psi})y_1+\partial_w y_1](1-bw)^{\rho_{\rm b}}}{y_1\partial_w y_2-y_2\partial_w y_1} \end{cases}$$

in which the denominator is a Wronskian identity (see Eq. (15.10.3) in [1]) that can be further simplified to:

$$y_1\partial_w y_2-y_2\partial_w y_1=b(1-\sigma_{\rm u}-\bar{\sigma}_{\rm b})(1-bw)^{\rho_\Delta w-1}(bw)^{-\sigma_{\rm u}-\bar{\sigma}_{\rm b}}.$$

Hence we have a complete general solution to the generating function $G(w,t)$. The solution is summarized as follows:

$$\boxed{\begin{aligned} w =&z-1 \\ \rho_\Delta =&\rho_{\rm b}-\rho_{\rm u} \\ \alpha+\beta =&\sigma_{\rm u}+\bar{\sigma}_{\rm b}-\rho_\Delta \\ \alpha\beta =&-\rho_\Delta\sigma_{\rm u} \\ f(w) =&\frac{(1-bw)^{\rho_{\rm b}}}{1-\sigma_{\rm u}-\bar{\sigma}_{\rm b}}[\rho_\Delta bw\,{}_2F_1(1-\rho_\Delta-\alpha,1-\rho_\Delta-\beta;2-\sigma_{\rm u}-\bar{\sigma}_{\rm b};bw) \\ &+(1-\sigma_{\rm u}-\bar{\sigma}_{\rm b})(1-bw)\,{}_2F_1(1-\rho_\Delta-\alpha,1-\rho_\Delta-\beta;1-\sigma_{\rm u}-\bar{\sigma}_{\rm b};bw)] \\ g(w) =&\frac{\rho_\Delta\sigma_{\rm b}(bw)^{\sigma_{\rm u}+\bar{\sigma}_{\rm b}}(1-bw)^{\rho_{\rm u}}}{(\bar{\sigma}_{\rm b}+\sigma_{\rm u})(\bar{\sigma}_{\rm b}+\sigma_{\rm u}-1)}\,{}_2F_1(\alpha,\beta;\sigma_{\rm u}+\bar{\sigma}_{\rm b}+1;bw) \\ G(w,t) =&f(we^{-t})\,{}_2F_1(\alpha,\beta;\sigma_{\rm u}+\bar{\sigma}_{\rm b};bw)(1-bw)^{-\rho_{\rm b}} \\ &+g(we^{-t})(bw)^{1-\sigma_{\rm u}-\bar{\sigma}_{\rm b}}\,{}_2F_1(1-\rho_\Delta-\alpha,1-\rho_\Delta-\beta;2-\sigma_{\rm u}-\bar{\sigma}_{\rm b};bw)(1-bw)^{-\rho_{\rm b}}. \end{aligned}} \tag{5}$$

Note that when $t \to \infty$, $f(w\mathrm{e}^{-t}) \to 1$ and $g(w\mathrm{e}^{-t}) \to 0$, and it yields the steady-state solution:

$$\boxed{G(w) = (1-bw)^{-\rho_\mathrm{b}}{}_2F_1(\alpha, \beta; \sigma_\mathrm{u} + \bar{\sigma}_\mathrm{b}; bw).}$$

The distribution of protein numbers is hence given by:

$$P(n_\mathrm{p}, t) = \frac{1}{n_\mathrm{p}!}\frac{\mathrm{d}^{n_\mathrm{p}}}{\mathrm{d}w^{n_\mathrm{p}}}G(w,t)|_{w=-1}. \tag{6}$$

Supplementary Note 2 Exact solution for linear GRN with oscillating transcription

The corresponding generating functions for the linear GRN with oscillating transcription are of the form:

$$\begin{cases} \partial_t G_0 + w\partial_w G_0 = \rho_\mathrm{u}\ell_t w G_0 + \sigma_\mathrm{u} G_1 - \bar{\sigma}_\mathrm{b} G_0, \\ \partial_t G_1 + w\partial_w G_1 = \rho_\mathrm{b}\ell_t w G_1 - \sigma_\mathrm{u} G_1 + \bar{\sigma}_\mathrm{b} G_0, \end{cases} \tag{7}$$

where ℓ_t is an oscillating function of the form $\ell_t = 1 + \mathrm{Am}\cos(k\pi t)$.
We define two quantities:

$$\mathbb{E}_0 = \exp\left(\rho_\mathrm{u} w\mathrm{e}^{-t}\int_0^t \ell_\tau \mathrm{e}^\tau \mathrm{d}\tau\right) \quad \text{and} \quad \mathbb{E}_1 = \exp\left(\rho_\mathrm{b} w\mathrm{e}^{-t}\int_0^t \ell_\tau \mathrm{e}^\tau \mathrm{d}\tau\right),$$

and let $G_0 = \mathbb{E}_1\tilde{G}_0$ and $G_1 = \mathbb{E}_1\tilde{G}_1$, which makes Eq. (7) equivalent to:

$$\begin{cases} \partial_t \tilde{G}_0 + w\partial_w \tilde{G}_0 = -\rho_\Delta\ell_t w\tilde{G}_0 + \sigma_\mathrm{u}\tilde{G}_1 - \bar{\sigma}_\mathrm{b}\tilde{G}_0, \\ \partial_t \tilde{G}_1 + w\partial_w \tilde{G}_1 = -\sigma_\mathrm{u}\tilde{G}_1 + \bar{\sigma}_\mathrm{b}\tilde{G}_0. \end{cases} \tag{8}$$

Solving $\tilde{G}_0$ from the second equation and plugging into the first equation, it is straightforward to obtain a single second-order partial differential equation for $\tilde{G}_1$:

$$\partial^2_{tt}\tilde{G}_1 + 2w\partial^2_{wt}\tilde{G}_1 + w^2\partial^2_{ww}\tilde{G}_1 + (\rho_\Delta\ell_t w + \sigma_\mathrm{u} + \bar{\sigma}_\mathrm{b})\partial_t\tilde{G}_1 + (\rho_\Delta\ell_t w + \sigma_\mathrm{u} + \bar{\sigma}_\mathrm{b} + 1)w\partial_w\tilde{G}_1 + \sigma_\mathrm{u}\rho_\Delta\ell_t w\tilde{G}_1 = 0.$$

By means of the transformation $u = \ln w - t$ and $v = w$, we obtain the compact form:

$$v\partial^2_{vv}\tilde{G}_1 + (\rho_\Delta\ell_t v + \Sigma)\partial_v\tilde{G}_1 + \sigma_\mathrm{u}\rho_\Delta\ell_t\tilde{G}_1 = 0,$$

where we used $\Sigma = 1 + \sigma_\mathrm{u} + \bar{\sigma}_\mathrm{b}$. The general solution of the above equation takes the form:

$$\tilde{G}_1 = \mathcal{F}_1(u)y_1 + \mathcal{G}_1(u)y_2,$$

where y_1 and y_2 are defined as:

$$\begin{cases} y_1 = M(\sigma_\mathrm{u}, \Sigma, x), \\ y_2 = (-\rho_\Delta\ell_t w)^{1-\Sigma}M(-\bar{\sigma}_\mathrm{b}, 2-\Sigma, x), \end{cases}$$

and $x = -\rho_\Delta\ell_t w$. Therefore, we can obtain $\tilde{G}_0$ from Eq. (8):

$$\tilde{G}_0 = \bar{\sigma}_\mathrm{b}^{-1}[-\rho_\Delta w(\ell_t + \dot{\ell}_t)(\mathcal{F}_1\partial_x y_1 + \mathcal{G}_1\partial_x y_2) + \sigma_\mathrm{u}(\mathcal{F}_1 y_1 + \mathcal{G}_1 y_2)]. \tag{9}$$

If we suppose that the initial condition is $G_0(w,0) = 1$ and $G_1(w,0) = 0$ (zero protein in promoter state

G), then we can solve for $\mathcal{F}_1$ and $\mathcal{G}_1$ from the following equations:

$$\begin{cases} \mathcal{F}_1 y_{1|0} + \mathcal{G}_1 y_{2|0} = 0 \\ [(-\rho_\Delta w \ell_0)\partial_x y_{1|0} + \sigma_\mathrm{u} y_{1|0}]\mathcal{F}_1 + [(-\rho_\Delta w \ell_0)\partial_x y_{2|0} + \sigma_\mathrm{u} y_{2|0}]\mathcal{G}_1 = \bar{\sigma}_\mathrm{b}, \end{cases}$$

where $y_{1|0}$ and $y_{2|0}$ are short hand for $y_1|_{t=0}$ and $y_2|_{t=0}$. This leads to:

$$\begin{cases} \mathcal{F}_1 = \dfrac{\bar{\sigma}_\mathrm{b} y_{2|0}}{[(-\rho_\Delta w \ell_0)\partial_x y_{1|0} + \sigma_\mathrm{u} y_{1|0}]y_{2|0} - [(-\rho_\Delta w \ell_0)\partial_x y_{2|0} + \sigma_\mathrm{u} y_{2|0}]y_{1|0}}, \\ \mathcal{G}_1 = -\dfrac{\bar{\sigma}_\mathrm{b} y_{1|0}}{[(-\rho_\Delta w \ell_0)\partial_x y_{1|0} + \sigma_\mathrm{u} y_{1|0}]y_{2|0} - [(-\rho_\Delta w \ell_0)\partial_x y_{2|0} + \sigma_\mathrm{u} y_{2|0}]y_{1|0}}. \end{cases}$$

Taking advantage of Wronskian identity, we have:

$$\begin{cases} \mathcal{F}_1(w) = \dfrac{\bar{\sigma}_\mathrm{b}}{\bar{\sigma}_\mathrm{b} + \sigma_\mathrm{u}} \exp(\ell_0 \rho_\Delta w) M(-\bar{\sigma}_\mathrm{b}, 2 - \Sigma, -\ell_0 \rho_\Delta w), \\ \mathcal{G}_1(w) = -\dfrac{\bar{\sigma}_\mathrm{b}}{\bar{\sigma}_\mathrm{b} + \sigma_\mathrm{u}} (-\ell_0 \rho_\Delta w)^{\bar{\sigma}_\mathrm{b} + \sigma_\mathrm{u}} \exp(\ell_0 \rho_\Delta w) M(\sigma_\mathrm{u}, \Sigma, -\rho_\Delta \ell_0 w), \end{cases}$$

and this completes the solution for G_1.

Next we obtain the solution for G_0 in an analogous manner. Let $G_0 = \mathbb{E}_0 \tilde{G}_0$ and $G_1 = \mathbb{E}_0 \tilde{G}_1$, which makes Eq. (7) equivalent to:

$$\begin{cases} \partial_t \tilde{G}_0 + w \partial_w \tilde{G}_0 = \sigma_\mathrm{u} \tilde{G}_1 - \bar{\sigma}_\mathrm{b} \tilde{G}_0, \\ \partial_t \tilde{G}_1 + w \partial_w \tilde{G}_1 = \rho_\Delta \ell_t w \tilde{G}_1 - \sigma_\mathrm{u} \tilde{G}_1 + \bar{\sigma}_\mathrm{b} \tilde{G}_0. \end{cases}$$

This equations can be used to derive a single partial differential equation for $\tilde{G}_0$:

$$v \partial^2_{vv} \tilde{G}_0 + (\sigma_\mathrm{u} + \bar{\sigma}_\mathrm{b} + 1 - \rho_\Delta \ell_t v)\partial_v G_0 - \bar{\sigma}_\mathrm{b} \rho_\Delta \ell_t \tilde{G}_0 = 0,$$

whose general solution is of the type:

$$\tilde{G}_0 = \mathcal{F}_0(u) y_1 + \mathcal{G}_0(u) y_2,$$

where y_1 and y_2 are defined as:

$$\begin{cases} y_1 = M(\bar{\sigma}_\mathrm{b}, \Sigma, \rho_\Delta \ell_t w) \\ y_2 = (\rho_\Delta \ell_t w)^{-\bar{\sigma}_\mathrm{b} - \sigma_\mathrm{u}} M(-\sigma_\mathrm{u}, 2 - \Sigma, \rho_\Delta \ell_t w). \end{cases}$$

Analogously to before, making use of the initial conditions enables us to derive expressions for the functions $\mathcal{F}_0$ and $\mathcal{G}_0$:

$$\begin{cases} \mathcal{F}_0(w) = \dfrac{\sigma_\mathrm{u}}{\bar{\sigma}_\mathrm{b} + \sigma_\mathrm{u}} \exp(-\ell_0 \rho_\Delta w) M(1 - \sigma_\mathrm{u}, 2 - \Sigma, \ell_0 \rho_\Delta w), \\ \mathcal{G}_0(w) = \dfrac{\bar{\sigma}_\mathrm{b}}{\bar{\sigma}_\mathrm{b} + \sigma_\mathrm{u}} (\ell_0 \rho_\Delta w)^{\bar{\sigma}_\mathrm{b} + \sigma_\mathrm{u}} \exp(-\ell_0 \rho_\Delta w) M(\bar{\sigma}_\mathrm{b} + 1, \Sigma, \ell_0 \rho_\Delta w). \end{cases}$$

Hence the solution for G_0 is now complete. Summarizing the full solution is as follows:

$$
\boxed{
\begin{aligned}
w =& z-1\\
\rho_\Delta =& \rho_\mathrm{b}-\rho_\mathrm{u}\\
\Sigma =& \bar{\sigma}_\mathrm{b}+\sigma_\mathrm{u}+1\\
\ell_t =& 1+\mathrm{Am}\cos(k\pi t)\\
\ell_0 =& 1+\mathrm{Am}\\
\mathbb{E}_0 =& \exp\left\{-\left(1+\frac{\mathrm{Am}}{1+k^2\pi^2}\right)\rho_\mathrm{u} w\mathrm{e}^{-t}+\rho_\mathrm{u} w\left[1+\frac{\cos(k\pi t)+k\pi\sin(k\pi t)}{1+k^2\pi^2}\mathrm{Am}\right]\right\}\\
\mathbb{E}_1 =& \exp\left\{-\left(1+\frac{\mathrm{Am}}{1+k^2\pi^2}\right)\rho_\mathrm{b} w\mathrm{e}^{-t}+\rho_\mathrm{b} w\left[1+\frac{\cos(k\pi t)+k\pi\sin(k\pi t)}{1+k^2\pi^2}\mathrm{Am}\right]\right\}\\
\mathcal{F}_0(w) =& \frac{\sigma_\mathrm{u}}{\bar{\sigma}_\mathrm{b}+\sigma_\mathrm{u}}\exp(-\ell_0\rho_\Delta w)M(1-\sigma_\mathrm{u},2-\Sigma,\ell_0\rho_\Delta w)\\
\mathcal{G}_0(w) =& \frac{\bar{\sigma}_\mathrm{b}}{\bar{\sigma}_\mathrm{b}+\sigma_\mathrm{u}}(\ell_0\rho_\Delta w)^{\bar{\sigma}_\mathrm{b}+\sigma_\mathrm{u}}\exp(-\ell_0\rho_\Delta w)M(\bar{\sigma}_\mathrm{b}+1,\Sigma,\ell_0\rho_\Delta w)\\
\mathcal{F}_1(w) =& \frac{\bar{\sigma}_\mathrm{b}}{\bar{\sigma}_\mathrm{b}+\sigma_\mathrm{u}}\exp(\ell_0\rho_\Delta w)M(-\bar{\sigma}_\mathrm{b},2-\Sigma,-\ell_0\rho_\Delta w)\\
\mathcal{G}_1(w) =& -\frac{\bar{\sigma}_\mathrm{b}}{\bar{\sigma}_\mathrm{b}+\sigma_\mathrm{u}}(-\ell_0\rho_\Delta w)^{\bar{\sigma}_\mathrm{b}+\sigma_\mathrm{u}}\exp(\ell_0\rho_\Delta w)M(\sigma_\mathrm{u},\Sigma,-\rho_\Delta\ell_0 w)\\
G_0(w,t) =& \mathbb{E}_0\left[\mathcal{F}_0(w\mathrm{e}^{-t})M(\bar{\sigma}_\mathrm{b},\Sigma,\rho_\Delta\ell_t w)+\mathcal{G}_0(w\mathrm{e}^{-t})(\rho_\Delta\ell_t w)^{-\sigma_\mathrm{u}-\bar{\sigma}_\mathrm{b}}M(-\sigma_\mathrm{u},2-\Sigma,\rho_\Delta\ell_t w)\right]\\
G_1(w,t) =& \mathbb{E}_1\left[\mathcal{F}_1(w\mathrm{e}^{-t})M(\sigma_\mathrm{u},\Sigma,-\rho_\Delta\ell_t w)+\mathcal{G}_1(w\mathrm{e}^{-t})(-\rho_\Delta\ell_t w)^{-\sigma_\mathrm{u}-\bar{\sigma}_\mathrm{b}}M(-\bar{\sigma}_\mathrm{b},2-\Sigma,-\rho_\Delta\ell_t w)\right]\\
G(w,t) =& G_0(w,t)+G_1(w,t).
\end{aligned}
}
\tag{10}
$$

As $t\to\infty$, the steady-state solution becomes:

$$
\boxed{
\begin{aligned}
G(w) =& \frac{\sigma_\mathrm{u} M(\bar{\sigma}_\mathrm{b},\Sigma,\rho_\Delta\ell_t w)}{\bar{\sigma}_\mathrm{b}+\sigma_\mathrm{u}}\exp\left[\rho_\mathrm{u} w\left(1+\frac{\cos(k\pi t)+k\pi\sin(k\pi t)}{1+k^2\pi^2}\mathrm{Am}\right)\right]\\
&+\frac{\bar{\sigma}_\mathrm{b} M(\sigma_\mathrm{u},\Sigma,-\rho_\Delta\ell_t w)}{\bar{\sigma}_\mathrm{b}+\sigma_\mathrm{u}}\exp\left[\rho_\mathrm{b} w\left(1+\frac{\cos(k\pi t)+k\pi\sin(k\pi t)}{1+k^2\pi^2}\mathrm{Am}\right)\right].
\end{aligned}
}
$$

The distribution of protein numbers is hence given by:

$$P(n_\mathrm{p},t)=\frac{1}{n_\mathrm{p}!}\frac{\mathrm{d}^{n_\mathrm{p}}}{\mathrm{d}w^{n_\mathrm{p}}}G(w,t)|_{w=-1}. \tag{11}$$

Furthermore, the mean of protein numbers at steady state is:

$$\boxed{\langle n_\mathrm{p}\rangle=\frac{\rho_\mathrm{b}\bar{\sigma}_\mathrm{b}+\rho_\mathrm{u}\sigma_\mathrm{u}}{\bar{\sigma}_\mathrm{b}+\sigma_\mathrm{u}}\left[1+\frac{\cos(k\pi t-\phi)}{\sqrt{1+k^2\pi^2}}\right],} \tag{12}$$

where $\phi=\arctan k\pi$.

Supplementary Note 3 Numerical evaluation of the LMA probability distributions

The numerical evaluation of the probability distribution solutions requires taking the nth derivative of the closed-form expressions for the generating functions which contain Kummer or hypergeometric functions. In principle, the derivatives can be computed directly using Mathematica's "D" function or similar functions in other symbolic computation software. However in practice this is found to be computation-

ally expensive and also leads to numeric instabilities particularly when the order of the derivatives is high. A way to circumvent these troubles leading to a high speed, accurate evaluation of the distribution is as follows.

Let the generating function be $G(z) = \sum_z z^n P(n)$. The Taylor series of this function about $z = 0$ is $G(z) = \sum_{i=0}^{\infty} a_i z^i$. It then follows by the definition of the probability distribution that:

$$P(n) = \frac{1}{n!} \frac{d^n}{dz^n} G(z)|_{z=0} = a_n. \tag{13}$$

Hence for computation of $P(n)$ there is no need to take the derivatives. Rather one first expands the generating function as a Taylor series using a symbolic computation software and then $P(n)$ is simply given by the nth coefficient of this series. In Mathematica this straightforwardly computed using the commands "Series" and "Series Coefficient", respectively. A Mathematica worksheet using this method for the computation of the probability distribution of the feedback loop, for all times, is provided (See Methods Code Availability in the main text).

In Supplementary Fig. 2 we quantify the speed of our method versus two common algorithms: the Finite State Projection algorithm [2] and the Stochastic Simulation Algorithm (SSA) [3]. The CPU times are measured on a laptop with an Intel i7 (2.6 GHz) quad-core processor. It is found that FSP's CPU time increases rapidly with the time t at which the probability distribution is evaluated and also significantly with the size of the truncated protein space. In contrast LMA's CPU time shows a very weak increase with t and is barely affected by the size of the protein space (see Supplementary Fig. 2A and 2B for details). For example, for the feedback loop with no cooperativity, the evaluation of probability distribution at time $t = 6$ takes about 1.5 mins using FSP whereas it takes about 2 seconds with the LMA (see Supplementary Fig. 2B). FSP's considerable computational time here stems from it solving a system of 600 equations, since the minimal truncation of the protein space for an accurate distribution evaluation is $[0, 300]$ and there are two promoter states. The LMA's computational time in contrast stems mostly from the evaluation of the effective binding rate which involves numerical integration over the interval $[0, t]$. In Supplementary Fig 2C we show the CPU time associated with computing the distribution by means of the SSA as a function of the number of realizations; to obtain an "acceptable" distribution, it clearly takes of the order of minutes whereas the LMA and FSP for this example take about 0.5 seconds and 2 seconds, respectively. Hence it is amply clear that that computation of the LMA can be done efficiently and involves a CPU time which is typically far less than that of FSP and SSA.

Supplementary Note 4 Exact solution for first-passage time of promoter switching in feedback loop in steady-state conditions

The calculation will be in two parts. First we calculate the first-passage time distribution to switch from G to $G^\star$ given the general initial condition of n proteins and promoter state G. Following we will use this result together with the marginal protein distribution in steady-state conditions to obtain the first-passage time of promoter switching (from G to $G^\star$).

For the purpose of calculating the first part of the calculation we only need to consider the following set of reactions:

$$G \xrightarrow{\rho_u} G + P, \quad P \xrightarrow{1} \varnothing, \quad G + P \xrightarrow{\sigma_b} G^\star. \tag{14}$$

The associated CMEs are:

$$\frac{dP(n_p, t)}{dt} = \rho_u[P(n_p - 1, t) - P(n_p, t)] + [(n_p + 1)P(n_p + 1, t) - n_p P(n_p, t)] - \sigma_b n_p P(n_p, t), \tag{15}$$

with $P(n_p, t)$ being the probability of observing n_p proteins and the promoter is in state G at time t. The general deterministic initial condition of n proteins and promoter state G is here assumed.

Multiplying z^{n_p} on both sides of Eq. (15) and summing over all n_p, we obtain the PDE that governs

the evolution of the generating function $G(z,t)$:

$$\partial_t G(z,t) = \rho_u (z-1) G(z,t) + [1-(1+\sigma_b)z]\partial_z G(z,t),$$

together with the initial condition $G(z,0) = z^n$.

Making use of the method of characteristics, the PDE problem is transformed into a set of coupled ODEs:

$$\begin{cases} \dfrac{dt}{ds} =1, \quad t(0)=0, \\ \dfrac{dz}{ds} =(1+\sigma_b)z-1, \quad z(0)=r, \\ \dfrac{dG}{ds} =\rho_u(z-1)G, \quad G(0)=r^n, \end{cases}$$

with r being a variable representing initial conditions. Solving these ODEs simultaneously, one obtains:

$$t=s, \quad z=\frac{[r(1+\sigma_b)-1]e^{(1+\sigma_b)s}+1}{1+\sigma_b}, \quad G=C(r)\exp\left[\frac{\rho_u[r(1+\sigma_b)-1]e^{(1+\sigma_b)s}}{(1+\sigma_b)^2} - \frac{\rho_u\sigma_b s}{1+\sigma_b}\right], \tag{16}$$

where

$$C(r) = r^n \exp\left[\frac{\rho_u[1-r(1+\sigma_b)]}{(1+\sigma_b)^2}\right].$$

Solving for r and s in terms of z and t from Eq. (16), the full solution of G is found to be:

$$G(z,t) = \left[\left(z-\frac{1}{1+\sigma_b}\right)e^{-(1+\sigma_b)t} + \frac{1}{1+\sigma_b}\right]^n \exp\left[\frac{\rho_u}{1+\sigma_b}\left(-\sigma_b t + \left(z-\frac{1}{1+\sigma_b}\right)\left(1-e^{-(1+\sigma_b)t}\right)\right)\right].$$

Hence the probability that at time t the system is still in state G is given by $G(1,t) = \sum_{n_p} P(n_p,t)$. The FPT distribution to switch from $G \to G^\star$ is then given by [4]:

$$P(t_{FP}=t|n_p=n, t=0) = -\partial_t G(1,t). \tag{17}$$

Now we come to the second and last part of the calculation. According to Bayes' theorem, the probability of FPT of the switch from G to $G^\star$ at steady-state can be computed via:

$$P(t_{FP}=t) = \sum_n \underbrace{P(t_{FP}=t|n_p=n,t=0)}_{A}\,\underbrace{P(n_p=n|n_g=0)}_{B}, \tag{18}$$

where part A corresponds to Eq. (17) and part B is the steady-state protein distribution conditional on the promoter being in the state G^* of the feedback loop (as given in [5]). Note that to calculate part B there are in principle three possible choices for the protein distribution: $P(n_p=n)$, $P(n_p=n|n_g=0)$ and $P(n_p=n|n_g=1)$. We use $P(n_p=n|n_g=0)$ because this is the distribution of proteins just before the switch from $G^\star$ to G, i.e., the distribution of proteins seen by the system when the reaction $G+P \to G^\star$ becomes first possible. A numerical validation of this intuition is presented in Supplementary Fig. 3. Note that while part A is computed using the subnetwork of the feedback loop (shown in schematic (14)), part B is computed using the full feedback loop; this is since part B requires knowledge of the steady-state conditional distribution of protein numbers which is determined by the back and forth switching between the two promoter states.

The distribution can also be computed approximately using the LMA. The approximation to Part A is calculated using the LMA as detailed in Methods 4.6 in the main text. The approximation to Part B is calculated from the LMA according to the recipe in Methods 4.2 in the main text.

Supplementary Note 5 LMA for feedback loop with Michaelis-Menten degradation in steady-state conditions

The feedback loop of with Michaelis-Menten (MM) protein degradation is given by:

$$G \xrightarrow{\rho_\mathrm{u}} G+P, \quad G+P \xrightarrow{\sigma_\mathrm{b}} G^\star, \quad G^\star \xrightarrow{\sigma_\mathrm{u}} G, \quad P \xrightarrow{J(n)} \varnothing,$$

in which $J(n_\mathrm{p}) = \frac{k_\mathrm{d} n_\mathrm{p}}{K+n_\mathrm{p}}$ is the MM degradation rate which is compatible with enzymatic degradation of the protein under quasi-equilibrium conditions. The LMA maps the nonlinear GRN to the corresponding "linear" GRN of the type:

$$G \xrightarrow{\rho_\mathrm{u}} G+P, \quad G \xrightarrow{\bar{\sigma}_\mathrm{b}} G^\star, \quad G^\star \xrightarrow{\sigma_\mathrm{u}} G, \quad P \xrightarrow{J(n)} \varnothing, \tag{19}$$

We now first solve the steady-state marginal protein distribution of the linear GRN and then use the LMA recipe to obtain the effective binding rate $\bar{\sigma}_\mathrm{b}$ in terms of the parameters of the original nonlinear GRN.

Steady-state solution of the linear mapped GRN

The CMEs describing (19) at steady-state are given by:

$$\rho_\mathrm{u}[P_0(n_\mathrm{p}-1) - P_0(n_\mathrm{p})] + J(n_\mathrm{p}+1)P_0(n_\mathrm{p}+1) - J(n_\mathrm{p})P_0(n_\mathrm{p}) - \bar{\sigma}_\mathrm{b} P_0(n_\mathrm{p}) + \sigma_\mathrm{u} P_1(n_\mathrm{p}) = 0, \tag{20}$$

and

$$J(n_\mathrm{p}+1)P_1(n_\mathrm{p}+1) - J(n_\mathrm{p})P_1(n_\mathrm{p}) + \bar{\sigma}_\mathrm{b} P_0(n_\mathrm{p}) - \sigma_\mathrm{u} P_1(n_\mathrm{p}) = 0, \tag{21}$$

where $P_0(n_\mathrm{p})$ and $P_1(n_\mathrm{p})$ are the probability that there are n_p proteins when the promoter is in state G and G^*, respectively. Adding together Eq. (20) and Eq. (21) gives:

$$J(n_\mathrm{p}+1)P(n_\mathrm{p}+1) - J(n_\mathrm{p})P(n_\mathrm{p}) + \rho_\mathrm{u}[P_0(n_\mathrm{p}-1) - P_0(n_\mathrm{p})] = 0. \tag{22}$$

with $P(n_\mathrm{p}) = P_0(n_\mathrm{p}) + P_1(n_\mathrm{p})$. Furthermore taking the summing of Eq. (22) from $n_\mathrm{p} = 0$ to N, and then setting $N = n_\mathrm{p}$, one obtains:

$$J(n_\mathrm{p}+1)P(n_\mathrm{p}+1) = \rho_\mathrm{u} P_0(n_\mathrm{p}),$$

which is tantamount to:

$$k_\mathrm{d}(n_\mathrm{p}+1)P(n_\mathrm{p}+1) = (K+1)\rho_\mathrm{u} P_0(n_\mathrm{p}) + \rho_\mathrm{u} n_\mathrm{p} P_0(n_\mathrm{p}). \tag{23}$$

The corresponding generating function of Eq. (23) is:

$$k_\mathrm{d}\partial_z G = \rho_\mathrm{u}(K+1)G_0 + \rho_\mathrm{u} z \partial_z G_0, \tag{24}$$

with $G(z) = \sum_i z^i P(i)$ and $G_j(z) = \sum_i z^i P_j(i)$ for $j = 0, 1$. It is noted that no assumption has been made hitherto.

Next we assume that:

$$J(n_\mathrm{p}+1) \approx \frac{k_\mathrm{d}(n_\mathrm{p}+1)}{K+n_\mathrm{p}},$$

an approximation that is expected to be good when the protein number is sufficiently large. The CME Eq. (21) is thus approximated by:

$$k_\mathrm{d}(n_\mathrm{p}+1)P_1(n_\mathrm{p}+1) - k_\mathrm{d} n_\mathrm{p} P_1(n_\mathrm{p}) + (K+n_\mathrm{p})[\bar{\sigma}_\mathrm{b} P_0(n_\mathrm{p}) - \sigma_\mathrm{u} P_1(n_\mathrm{p})] = 0,$$

which has the associated generating function equation:

$$k_{\rm d}(1-z)\partial_z G_1 + \bar{\sigma}_{\rm b} K G_0 + \bar{\sigma}_{\rm b} z\partial_z G_0 - \sigma_{\rm u} K G_1 - \sigma_{\rm u} z\partial_z G_1 = 0. \tag{25}$$

Solving $\partial_z G_1$ from Eq. (24) together with $G = G_1 + G_0$, differentiating Eq. (25) with respect to z and combining the two, it follows that:

$$-\mathcal{L}G_0 + (\mathcal{M} - \mathcal{N}z)\partial_z G_0 + (-k_{\rm d}^2 + \mathcal{P}z - \mathcal{Q}z^2)\partial_{zz}^2 G_0 = 0, \tag{26}$$

where

$$\begin{aligned}
\mathcal{L} =& (1+K)\rho_{\rm u}(k_{\rm d} + \sigma_{\rm u} + K\sigma_{\rm u}),\\
\mathcal{M} =& k_{\rm d}[k_{\rm d} + (2+K)\rho_{\rm u} + (1+K)(\bar{\sigma}_{\rm b} + \sigma_{\rm u})],\\
\mathcal{N} =& \rho_{\rm u}[(3+K)k_{\rm d} + (3+2K)\sigma_{\rm u}],\\
\mathcal{P} =& k_{\rm d}(k_{\rm d} + \rho_{\rm u} + \bar{\sigma}_{\rm b} + \sigma_{\rm u}),\\
\mathcal{Q} =& \rho_{\rm u}(k_{\rm d} + \sigma_{\rm u}).
\end{aligned}$$

To transform Eq. (26) into the following standard form of hypergeometric function ${}_2F_1(\alpha, \beta, \gamma, w)$:

$$w(1-w)\partial_{ww}^2 G_0 + (\gamma - (\alpha+\beta+1)w)\partial_w G_0 - \alpha\beta G_0 = 0,$$

we apply the variable transformation $z = \mathcal{A}w + \mathcal{B}$ with $\mathcal{A}$ and $\mathcal{B}$ as undetermined real numbers. Hence, Eq. (26) becomes:

$$\mathcal{A}^{-2}[-k_{\rm d}^2 + \mathcal{P}\mathcal{B} - \mathcal{Q}\mathcal{B}^2 + (\mathcal{P}\mathcal{A} - 2\mathcal{Q}\mathcal{A}\mathcal{B})w - \mathcal{Q}\mathcal{A}^2 w^2]\partial_{ww}^2 G_0 + \mathcal{A}^{-1}(\mathcal{M} - \mathcal{N}\mathcal{B} - \mathcal{N}\mathcal{A}w)\partial_w G_0 - \mathcal{L}G_0 = 0,$$

which further implies that $\mathcal{A}$ and $\mathcal{B}$ can be solved from the simultaneous equations:

$$\begin{cases} -k_{\rm d}^2 + \mathcal{P}B - \mathcal{Q}\mathcal{B}^2 = 0,\\ \mathcal{P}\mathcal{A} - 2\mathcal{Q}\mathcal{A}\mathcal{B} = \mathcal{Q}\mathcal{A}^2. \end{cases}$$

Thus, the generating function G_0 can be represented as:

$$G_0 = \Lambda \times {}_2F_1\left(\alpha, \beta, \gamma, \frac{z-\mathcal{B}}{\mathcal{A}}\right),$$

in which α and β are solved from

$$\begin{cases} \alpha + \beta + 1 - \dfrac{\mathcal{N}}{\mathcal{Q}} = 0,\\ \alpha\beta - \dfrac{\mathcal{L}}{\mathcal{Q}} = 0, \end{cases}$$

$$\gamma = \frac{\mathcal{M} - \mathcal{N}\mathcal{B}}{\mathcal{Q}\mathcal{A}},$$

and Λ is the normalization constant.

The probability $P(n_{\rm p})$ is of more interest in practice and can be calculated from Eq. (24):

$$P(n_{\rm p}) = \frac{1}{n_{\rm p}!}\partial_z^{n_{\rm p}} G = \frac{1}{k_{\rm d} n_{\rm p}!}\partial_z^{n_{\rm p}-1}\left[\rho_{\rm u}(K+1)G_0 + \rho_{\rm u} z\partial_z G_0\right]. \tag{27}$$

for $n \geq 1$. Using Eq. (20) one can also obtain the probability $P(0)$ from the equation:

$$P(0) = \sigma_{\rm u}^{-1}[-(\rho_{\rm u} + \bar{\sigma}_{\rm b})P_0(0) + J(1)P_0(1)] + P_0(0).$$

The normalisation constant is difficult to obtain in closed form and is easiest computed numerically using $\Lambda = \left[\sum_{n_\mathrm{p}} P(n_\mathrm{p})\right]^{-1}$.

Computation of effective binding rate $\bar{\sigma}_\mathrm{b}$ using the LMA recipe

The steady-state moment equations of the reaction system (19) are:

$$M_\mathrm{ne}: \begin{cases} \rho_\mathrm{u}\langle n_\mathrm{g}\rangle - \left\langle \dfrac{k_\mathrm{d} n_\mathrm{p}}{K + n_\mathrm{p}} \right\rangle = 0, \\ -\bar{\sigma}_\mathrm{b}\langle n_\mathrm{g}\rangle + \sigma_\mathrm{u}(1 - \langle n_\mathrm{g}\rangle) = 0, \\ \rho_\mathrm{u}\langle n_\mathrm{g}\rangle - \left\langle \dfrac{k_\mathrm{d} n_\mathrm{p} n_\mathrm{g}}{K + n_\mathrm{p}} \right\rangle - \bar{\sigma}_\mathrm{b}\langle n_\mathrm{p} n_\mathrm{g}\rangle + \sigma_\mathrm{u}\langle n_\mathrm{p}\rangle - \sigma_\mathrm{u}\langle n_\mathrm{p} n_\mathrm{g}\rangle = 0. \end{cases}$$

Note that the set of equations M_ne has statistical averages taken over rational functions of n_p which leads to a set of non-closed moment equations. To make it closed, the following set of equations are used to get an approximate solution:

$$M_\mathrm{le}: \begin{cases} \rho_\mathrm{u}\langle n_\mathrm{g}\rangle - \dfrac{k_\mathrm{d}\langle n_\mathrm{p}\rangle}{K + \langle n_\mathrm{p}\rangle} = 0, \\ -\bar{\sigma}_\mathrm{b}\langle n_\mathrm{g}\rangle + \sigma_\mathrm{u}(1 - \langle n_\mathrm{g}\rangle) = 0, \\ \rho_\mathrm{u}\langle n_\mathrm{g}\rangle - \dfrac{k_\mathrm{d}\langle n_\mathrm{p} n_\mathrm{g}\rangle}{K + \langle n_\mathrm{p}\rangle} - \bar{\sigma}_\mathrm{b}\langle n_\mathrm{p} n_\mathrm{g}\rangle + \sigma_\mathrm{u}\langle n_\mathrm{p}\rangle - \sigma_\mathrm{u}\langle n_\mathrm{p} n_\mathrm{g}\rangle = 0 \end{cases}$$

Substituting $\bar{\sigma}_\mathrm{b} = \sigma_\mathrm{b}\langle n_\mathrm{p} n_\mathrm{g}\rangle / \langle n_\mathrm{g}\rangle$ in the above equations, we can solve for the moments $\langle n_\mathrm{p} n_\mathrm{g}\rangle$, $\langle n_\mathrm{p}\rangle$ and $\langle n_\mathrm{g}\rangle$. Finally using these moments to compute $\bar{\sigma}_\mathrm{b} = \sigma_\mathrm{b}\langle n_\mathrm{p} n_\mathrm{g}\rangle / \langle n_\mathrm{g}\rangle$ and substituting in the explicit solution Eq. (27) gives us the LMA steady-state distribution of protein numbers for the feedback loop with Michaelis-Menten degradation.

Supplementary Note 6 LMA describing the time-evolution of the toggle switch

The toggle switch system [6] consists of the following set of reactions:

$$\begin{aligned} &G_0 \xrightarrow{\rho_1} G_0 + P, \quad G_0^\star \xrightarrow{\rho_2} G_0^\star + P, \quad G_0 + M \underset{\sigma_2}{\overset{\sigma_1}{\rightleftharpoons}} G_0^\star, \quad P \xrightarrow{1} \varnothing, \\ &G_1 \xrightarrow{\rho_3} G_1 + M, \quad G_1^\star \xrightarrow{\rho_4} G_1^\star + M, \quad G_1 + P \underset{\sigma_4}{\overset{\sigma_3}{\rightleftharpoons}} G_1^\star, \quad M \xrightarrow{k} \varnothing. \end{aligned}$$

Two proteins P and M are expressed by two different genes and they act as transcriptional activators or repressors of the gene other than the one which expressed them. This is a typical example of a system of four coupled promoter states, namely (G_0, G_1), $(G_0^\star, G_1)$, $(G_0, G_1^\star)$ and $(G_0^\star, G_1^\star)$. Note that the we non-dimensionalise time and all rate parameters by the the degradation rate of protein P.

Suppose that we are interested in the marginal distributions of proteins of P and M at time t.

The nonlinear GRN above is mapped according to the LMA recipe to the linear GRN:

$$\begin{aligned} &G_0 \xrightarrow{\rho_1} G_0 + P, \quad G_0^\star \xrightarrow{\rho_2} G_0^\star + P, \quad G_0 \underset{\sigma_2}{\overset{\bar{\sigma}_1}{\rightleftharpoons}} G_0^\star, \quad P \xrightarrow{1} \varnothing, \\ &G_1 \xrightarrow{\rho_3} G_1 + M, \quad G_1^\star \xrightarrow{\rho_4} G_1^\star + M, \quad G_1 \underset{\sigma_4}{\overset{\bar{\sigma}_3}{\rightleftharpoons}} G_1^\star, \quad M \xrightarrow{k} \varnothing, \end{aligned} \tag{28}$$

where $\bar{\sigma}_1$ and $\bar{\sigma}_3$ are effective reaction rates to be determined. Note that these effective reaction rates $\bar{\sigma}_1$

and $\bar{\sigma}_3$ absorb the coupling between the two genes.

First we compute the effective binding rates $\bar{\sigma}_1$ and $\bar{\sigma}_3$ in terms of the rate constants of the original nonlinear GRN. The moment equations of (28) are given by:

$$\mathcal{M}_{\mathrm{TG}}(\bar{\sigma}_1,\bar{\sigma}_3):\begin{cases}\partial_t\langle n_{\mathrm{p}}\rangle =\rho_1\langle n_{\mathrm{g}_0}\rangle+\rho_2(1-\langle n_{\mathrm{g}_0}\rangle)-\langle n_{\mathrm{p}}\rangle,\\ \partial_t\langle n_{\mathrm{m}}\rangle =\rho_3\langle n_{\mathrm{g}_1}\rangle+\rho_4(1-\langle n_{\mathrm{g}_1}\rangle)-k\langle n_{\mathrm{m}}\rangle,\\ \partial_t\langle n_{\mathrm{g}_0}\rangle =-\bar{\sigma}_1\langle n_{\mathrm{g}_0}\rangle+\sigma_2(1-\langle n_{\mathrm{g}_0}\rangle),\\ \partial_t\langle n_{\mathrm{g}_1}\rangle =-\bar{\sigma}_3\langle n_{\mathrm{g}_1}\rangle+\sigma_4(1-\langle n_{\mathrm{g}_1}\rangle),\\ \partial_t\langle n_{\mathrm{p}}n_{\mathrm{g}_1}\rangle =-(1+\bar{\sigma}_3+\sigma_4)\langle n_{\mathrm{p}}n_{\mathrm{g}_1}\rangle+(\rho_1-\rho_2)\langle n_{\mathrm{g}_0}n_{\mathrm{g}_1}\rangle+\rho_2\langle n_{\mathrm{g}_1}\rangle+\sigma_4\langle n_{\mathrm{p}}\rangle,\\ \partial_t\langle n_{\mathrm{m}}n_{\mathrm{g}_0}\rangle =-(k+\bar{\sigma}_1+\sigma_2)\langle n_{\mathrm{m}}n_{\mathrm{g}_0}\rangle+(\rho_3-\rho_4)\langle n_{\mathrm{g}_0}n_{\mathrm{g}_1}\rangle+\rho_4\langle n_{\mathrm{g}_0}\rangle+\sigma_2\langle n_{\mathrm{m}}\rangle,\\ \partial_t\langle n_{\mathrm{g}_0}n_{\mathrm{g}_1}\rangle =-(\bar{\sigma}_1+\sigma_2+\bar{\sigma}_3+\sigma_4)\langle n_{\mathrm{g}_0}n_{\mathrm{g}_1}\rangle+\sigma_2\langle n_{\mathrm{g}_1}\rangle+\sigma_4\langle n_{\mathrm{g}_0}\rangle,\end{cases}\tag{29}$$

where $n_{\mathrm{p}}, n_{\mathrm{m}}$ are numbers of protein P and M respectively, $n_{\mathrm{g}_0}=1$ and $n_{\mathrm{g}_1}=1$ denote the gene state G_0 and G_1 respectively. We substitute $\bar{\sigma}_1=\sigma_1\langle n_{\mathrm{m}}n_{\mathrm{g}_0}\rangle/\langle n_{\mathrm{g}_0}\rangle$ and $\bar{\sigma}_3=\sigma_3\langle n_{\mathrm{p}}n_{\mathrm{g}_1}\rangle/\langle n_{\mathrm{g}_1}\rangle$ in the above moment equations and solve them on the time interval $t'\in[0,t]$. Denoting the solved moments of interest at time t' as $\langle n_{\mathrm{m}}n_{\mathrm{g}_0}\rangle_{t'}$, $\langle n_{\mathrm{p}}n_{\mathrm{g}_1}\rangle_{t'}$, $\langle n_{\mathrm{g}_0}\rangle_{t'}$ and $\langle n_{\mathrm{g}_1}\rangle_{t'}$, we have, by the LMA recipe, that the effective (time-averaged) reaction rates at time t are given by:

$$\sigma_1^*=\frac{\sigma_1}{t}\int_0^t\frac{\langle n_{\mathrm{m}}n_{\mathrm{g}_0}\rangle_{t'}}{\langle n_{\mathrm{g}_0}\rangle_{t'}}\mathrm{d}t',\quad \sigma_3^*=\frac{\sigma_3}{t}\int_0^t\frac{\langle n_{\mathrm{p}}n_{\mathrm{g}_1}\rangle_{t'}}{\langle n_{\mathrm{g}_1}\rangle_{t'}}\mathrm{d}t'.$$

Finally we determined the marginal distributions at time t as follows. The LMA decouples the toggle switch into two separate linear GRNs. This decoupling is a great advantage of the LMA since the solution of these two linear GRNs has already been given in the main text in the context of the linear GRN arising from the feedback loop. Thus we can immediately state that the marginal protein number distribution for P at time t is given by replacing $(\rho_{\mathrm{u}},\rho_{\mathrm{b}},\sigma_{\mathrm{u}},\bar{\sigma}_{\mathrm{b}})$ in Eq. (11-13) in the main text by $(\rho_1,\rho_2,\sigma_2,\bar{\sigma}_1^*)$. Similarly the marginal protein number distribution for M is given by replacing $(\rho_{\mathrm{u}},\rho_{\mathrm{b}},\sigma_{\mathrm{u}},\bar{\sigma}_{\mathrm{b}})$ in Eq. (11-13) in the main text by $(\rho_3/k,\rho_4/k,\sigma_4/k,\bar{\sigma}_3^*/k)$.

Supplementary Note 7 LMA for the feedback loop with explicit mRNA transcription and protein translation in steady-state conditions

This nonlinear GRN is an extension of the feedback loop where we now include explicitly the mRNA dynamics besides that of protein:

$$\begin{aligned}&G\xrightarrow{\rho_{\mathrm{u}}}G+M,\quad G^{\star}\xrightarrow{\rho_{\mathrm{b}}}G^{\star}+M,\quad M\xrightarrow{\rho}M+P,\\ &M\xrightarrow{\gamma}\varnothing,\quad P\xrightarrow{d}\varnothing,\quad G+P\underset{\sigma_{\mathrm{u}}}{\overset{\sigma_{\mathrm{b}}}{\rightleftharpoons}}G^{\star}.\end{aligned}$$

Here M and P are mRNA and protein respectively. The linear GRN that the above nonlinear GRN maps onto is the well-known three-stage model of gene expression [7]:

$$\begin{aligned}&G\xrightarrow{\rho_{\mathrm{u}}}G+M,\quad G^{\star}\xrightarrow{\rho_{\mathrm{b}}}G^{\star}+M,\quad M\xrightarrow{\rho}M+P,\\ &M\xrightarrow{\gamma}\varnothing,\quad P\xrightarrow{d}\varnothing,\quad G\underset{\sigma_{\mathrm{u}}}{\overset{\bar{\sigma}_{\mathrm{b}}}{\rightleftharpoons}}G^{\star},\end{aligned}\tag{30}$$

where $\bar{\sigma}_{\mathrm{b}}$ is an effective rate to be determined as follows. The moment equations of (30) are given by:

$$\begin{cases} \partial_t \langle n_{\mathrm{m}} \rangle = \rho_{\mathrm{u}} \langle n_{\mathrm{g}} \rangle + \rho_{\mathrm{b}} (1 - \langle n_{\mathrm{g}} \rangle) - \gamma \langle n_{\mathrm{m}} \rangle, \\ \partial_t \langle n_{\mathrm{p}} \rangle = \rho \langle n_{\mathrm{m}} \rangle - d \langle n_{\mathrm{p}} \rangle, \\ \partial_t \langle n_{\mathrm{g}} \rangle = - \bar{\sigma}_{\mathrm{b}} \langle n_{\mathrm{g}} \rangle + \sigma_{\mathrm{u}} (1 - \langle n_{\mathrm{g}} \rangle), \\ \partial_t \langle n_{\mathrm{p}} n_{\mathrm{g}} \rangle = \rho \langle n_{\mathrm{m}} n_{\mathrm{g}} \rangle - (d + \bar{\sigma}_{\mathrm{b}} + \sigma_{\mathrm{u}}) \langle n_{\mathrm{p}} n_{\mathrm{g}} \rangle + \sigma_{\mathrm{u}} \langle n_{\mathrm{p}} \rangle, \\ \partial_t \langle n_{\mathrm{m}} n_{\mathrm{g}} \rangle = \rho_{\mathrm{u}} \langle n_{\mathrm{g}} \rangle - (\sigma_{\mathrm{u}} + \bar{\sigma}_{\mathrm{b}} + \gamma) \langle n_{\mathrm{m}} n_{\mathrm{g}} \rangle + \sigma_{\mathrm{u}} \langle n_{\mathrm{m}} \rangle, \end{cases}$$

where we have use the fact that since n_{g} is a Boolean variable then $n_{\mathrm{g}}^2 = n_{\mathrm{g}}$. Substituting $\bar{\sigma}_{\mathrm{b}} = \sigma_{\mathrm{b}} \langle n_{\mathrm{p}} n_{\mathrm{g}} \rangle / \langle n_{\mathrm{g}} \rangle$ in the above equations and solving the resulting moment equations at steady-state, one obtains $\langle n_{\mathrm{p}} n_{\mathrm{g}} \rangle$ and $\langle n_{\mathrm{g}} \rangle$, from which one can construct the effective binding rate $\sigma_{\mathrm{b}}^* = \sigma_{\mathrm{b}} \frac{\langle n_{\mathrm{p}} n_{\mathrm{g}} \rangle}{\langle n_{\mathrm{g}} \rangle}$.

The steady-state marginal distribution of mRNA numbers for (30) is well known [7–9] and is given by Eqs. (14-15) in the main text with $(\rho_{\mathrm{u}}, \rho_{\mathrm{b}}, \bar{\sigma}_{\mathrm{b}}, \sigma_{\mathrm{u}})$ replaced by $(\rho_{\mathrm{u}}/\gamma, \rho_{\mathrm{b}}/\gamma, \sigma_{\mathrm{b}}^*/\gamma, \sigma_{\mathrm{u}}/\gamma)$.

The protein distribution cannot be obtained explicitly from LMA, as currently there is no known exact solution for protein distribution reported for the three-stage model of gene expression. However, all the moments of protein are obtainable since the moment equations are closed for the linear GRN (30).

Supplementary Note 8 Some proofs related to the time-averaging assumption

First term of the Magnus series of the master equation gives a well-defined probability vector

Consider the master equation:

$$\dot{\mathbf{P}}_{\mathrm{N}}(t) = \mathbf{A}_{\mathrm{L}}(t) \mathbf{P}_{\mathrm{N}}(t), \quad \mathbf{P}_{\mathrm{N}}(0) = \mathbf{P}_0 \tag{31}$$

in which $\mathbf{A}_{\mathrm{L}}(t)$ is a zero-column-sum matrix with negative diagonal elements and positive non-diagonal elements for all times t (these properties are natural to any master equation [2]). These properties guarantee that $\mathbf{P}_{\mathrm{N}}(t)$ is a probability vector at all times (the elements of $\mathbf{P}_{\mathrm{N}}(t)$ are greater than or equal to zero and they sum to 1 for all times). The proposed approximate solution at time $t = T$ is the first term of the Magnus expansion:

$$\mathbf{P}_{\mathrm{L}}(T) = \exp\left(\int_0^T \mathbf{A}_{\mathrm{L}}(t) \mathrm{d}t \right) \mathbf{P}_0.$$

We are interested in whether $\mathbf{P}_{\mathrm{L}}(T)$ is a well-defined probability vector or not. For convenience, we also define:

$$\mathbf{A}_{\mathrm{C,T}} = \frac{1}{T} \int_0^T \mathbf{A}_{\mathrm{L}}(t) \mathrm{d}t.$$

Claim 1: The matrix $\mathbf{A}_{\mathrm{C,T}}$ is a zero-column-sum matrix, all its diagonal elements are negative and all its non-diagonal elements are positive.

First, we divide T into N small intervals whose length is all Δ. On the i-th interval the matrix $\mathbf{A}_{\mathrm{L}}(t)$ is approximated by a constant matrix $\mathbf{A}_{\mathrm{L}}^i := \mathbf{A}_{\mathrm{L}}((i-1)\Delta)$, namely,

$$\mathbf{A}_{\mathrm{C,T}} \approx \frac{\sum_{i=1}^N \mathbf{A}_{\mathrm{L}}^i \Delta}{N\Delta} = \frac{\sum_{i=1}^N \mathbf{A}_{\mathrm{L}}^i}{N}.$$

Thus, the summation of the j-th column of $\mathbf{A}_{\mathrm{C,T}}$ is:

$$[\mathbf{A}_{\mathrm{C,T}}]_j \approx \frac{1}{N} \sum_{i=1}^N [\mathbf{A}_{\mathrm{L}}^i]_j = 0,$$

where $[\cdot]_j$ stands for the summation of all elements of the j-th column.The second equality holds because of zero-column-sum of $\mathbf{A}_\mathrm{L}^i$. Taking the limit $N \to \infty$ of the equation above leads to the zero-column-sum property of $\mathbf{A}_\mathrm{C,T}$. The diagonal element properties follow by similar reasoning.

Claim 2: The vector $\mathbf{P}_\mathrm{L}(T)$ is a probability vector with positive elements and the summation of which is equal to 1 for any time T.

It is noted that $\mathbf{P}_\mathrm{L}(T)$ is also a solution to the differential equation:

$$\dot{\mathbf{P}}_\mathrm{L}(t) = \mathbf{A}_\mathrm{C,T}\mathbf{P}_\mathrm{L}(t), \quad \mathbf{P}_\mathrm{L}(0) = \mathbf{P}_0$$

at time T. Note that this is possible because $\mathbf{A}_\mathrm{C,T}$ is a time-independent matrix. By the properties of $\mathbf{A}_\mathrm{C,T}$ in Claim 1, it follows that $\mathbf{P}_\mathrm{L}(T)$ is a probability vector. Hence it follows that the first term of the Magnus expansion has a meaningful interpretation in a probabilistic sense.

Exponential convergence of moments

The moment equations can always be written in the form $\dot{\mathbf{x}} = \mathbf{\Psi}(t)\mathbf{x} + \mathbf{b}$ where $\mathbf{x}$ is the moment vector, $\Psi(t)$ is Hurwitz uniformly in time and $\mathbf{b}$ is a constant vector. The proof below shows that since $\Psi(t)$ is Hurwitz uniformly in time, there exists a Lyapunov functional with the property that the time derivative of the Lyapunov functional is less than a negative constant times the functional itself; this then implies exponential convergence of the moment vector to a steady-state constant.

To discuss the stability of the $\dot{\mathbf{x}} = \mathbf{\Psi}(t)\mathbf{x} + \mathbf{b}$ it is enough to investigate the system $\dot{\mathbf{z}} = \mathbf{\Psi}(t)\mathbf{z}$ since the time dependence is not affected by the constant vector $\mathbf{b}$. For nonlinear gene regulatory networks with constant rates, the LMA leads to an approximately equivalent linear gene regulatory networks with a time-dependent protein-promoter binding rate $\bar{\sigma}_\mathrm{b}(t)$. Hence the time dependence of $\mathbf{\Psi}(t)$ is because of $\bar{\sigma}_\mathrm{b}(t)$. Now since $\bar{\sigma}_\mathrm{b}(t)$ is positive for all time, it is easy to check (for a given particular gene regulatory network) that $\mathbf{\Psi}(t)$ is Hurwitz for any time t, i.e., the real part of the eigenvalues of $\mathbf{\Psi}(t)$ is negative for all times. According to Lemma 9.9 in [10], there exists a positive definite solution $\mathbf{P}(\bar{\sigma}_\mathrm{b}(t))$ satisfying a Lyapunov equation:

$$\mathbf{P}(\bar{\sigma}_\mathrm{b}(t))\mathbf{\Psi}(t) + \mathbf{\Psi}^\top(t)\mathbf{P}(\bar{\sigma}_\mathrm{b}(t)) = -\mathbf{I}.$$

Told by the same lemma, $\|\partial_{\bar{\sigma}_\mathrm{b}(t)}\mathbf{P}(\bar{\sigma}_\mathrm{b}(t))\|$ is upper bounded by c_5 and $c_1\mathbf{z}^\top\mathbf{z} \le \mathbf{z}^\top\mathbf{P}(\bar{\sigma}_\mathrm{b}(t))\mathbf{z}$. Therefore, the derivative of Lyapunov functional $\mathbf{V}(t,\mathbf{z}) = \mathbf{z}^\top\mathbf{P}(\bar{\sigma}_\mathrm{b}(t))\mathbf{z}$ becomes:

$$\begin{aligned}\dot{\mathbf{V}}(t,\mathbf{z}) =& \mathbf{z}^\top[\mathbf{P}(\bar{\sigma}_\mathrm{b}(t))\mathbf{\Psi}(t) + \mathbf{\Psi}^\top(t)\mathbf{P}(\bar{\sigma}_\mathrm{b}(t)) + \dot{\mathbf{P}}(\bar{\sigma}_\mathrm{b}(t))]\mathbf{z} \\ =& \mathbf{z}^\top[-\mathbf{I} + \partial_{\bar{\sigma}_\mathrm{b}(t)}\mathbf{P}(\bar{\sigma}_\mathrm{b}(t)) \times \dot{\bar{\sigma}}_\mathrm{b}(t)]\mathbf{z} \\ \le& -(1 - c_5|\dot{\bar{\sigma}}_\mathrm{b}(t)|)\|\mathbf{z}\|^2.\end{aligned}$$

Now $\dot{\bar{\sigma}}_\mathrm{b}(t)$ is proportional to σ_b (follows by the definition of $\bar{\sigma}_\mathrm{b}(t)$ in the main text). Hence if σ_b is small enough, we have:

$$\dot{\mathbf{V}}(t,z) \le -\epsilon\|\mathbf{z}\|^2$$

for some positive real ϵ. By means of $c_1\mathbf{z}^\top\mathbf{z} \le \mathbf{z}^\top\mathbf{P}(\bar{\sigma}_\mathrm{b}(t))\mathbf{z}$, we have

$$\dot{\mathbf{V}}(t,\mathbf{z}) \le -(\epsilon/c_1)\mathbf{V}(t,\mathbf{z}).$$

By the Comparison Lemma (p.103 in [10]), there exists a system $\dot{\mathbf{W}} = -(\epsilon/c_1)\mathbf{W}$ which upper bounds $\mathbf{V}(t,\mathbf{z})$ for all the time and converges exponentially. This observation indicates the exponential convergence of the moments and it further implies that $\bar{\sigma}_\mathrm{b}(t)$ reaches the steady state value $\bar{\sigma}_\mathrm{b}(\infty)$ exponentially as well (follows by the definition of $\bar{\sigma}_\mathrm{b}(t)$ in the main text).

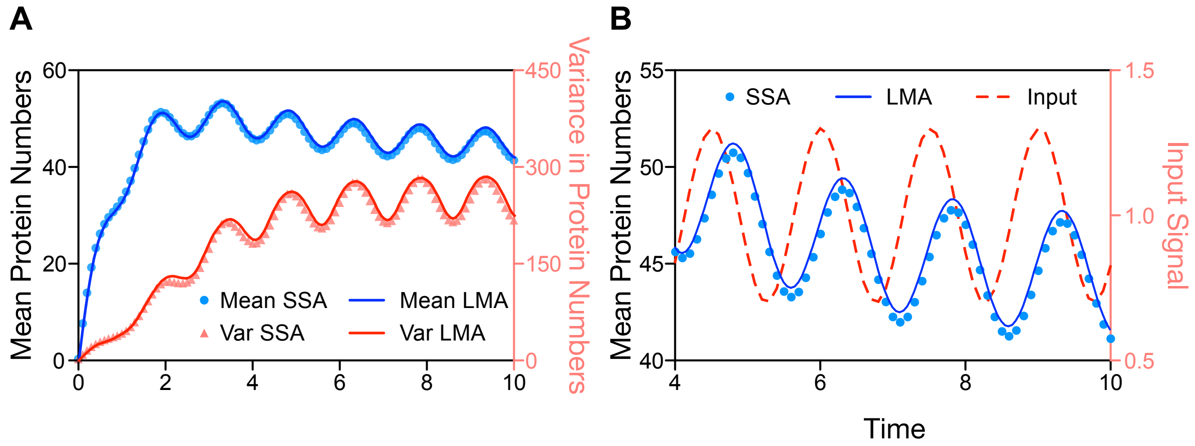

Supplementary Figure 1: Accuracy of the LMA approximation of the moments of the nonlinear GRN with oscillating transcription. The structure of the GRN is shown in Supplementary Fig. 2(C) of the main text. (A) a comparison of the mean and variance in protein numbers obtained from the LMA approximation and those obtained from the SSA. (B) compares the input signal $1 + \mathrm{Am}\cos(k\pi t)$ (modulating the transcription rate) with the mean protein numbers from the SSA and the LMA Eq. (12). The parameters are $\rho_{\mathrm{u}} = 60, \rho_{\mathrm{b}} = 25, \mathrm{Am} = 0.3, k = 1.33, \sigma_{\mathrm{b}} = 0.004, \sigma_{\mathrm{u}} = 0.25$. The phase differences between the input and the mean given by the SSA is 1.3322 rad, while the theoretical phase difference predicted by LMA is 1.3359 rad.

Type	(ρ_u, ρ_b)	Feedback Loop		Cooperativity		Protein Bursts	
		LMA	Num. Int.	LMA	Num. Int.	LMA	Num. Int.
Repressor	(60,25)	58.53	57.87	32.41	31.42	36.71	36.41
	(50,25)	35.76	35.52	17.31	16.93	16.24	16.21
	(40,25)	5.25	5.22	2.49	2.43	0.61	0.60
Activator	(25,60)	59.19	60.57	35.86	39.15	43.15	44.13
	(25,50)	36.85	37.32	20.84	21.98	18.82	19.13
	(25,40)	5.71	5.78	3.24	3.37	0.74	0.76

Supplementary Table 1: Table showing the percentage of the logarithmic space in Fig 9 (main text) where the steady-state distribution is bimodal, i.e., where there is noise-induced bistability, according to the LMA and to numerical integration ('Num. Int.") of the master equations of the three types of nonlinear feedback loops.

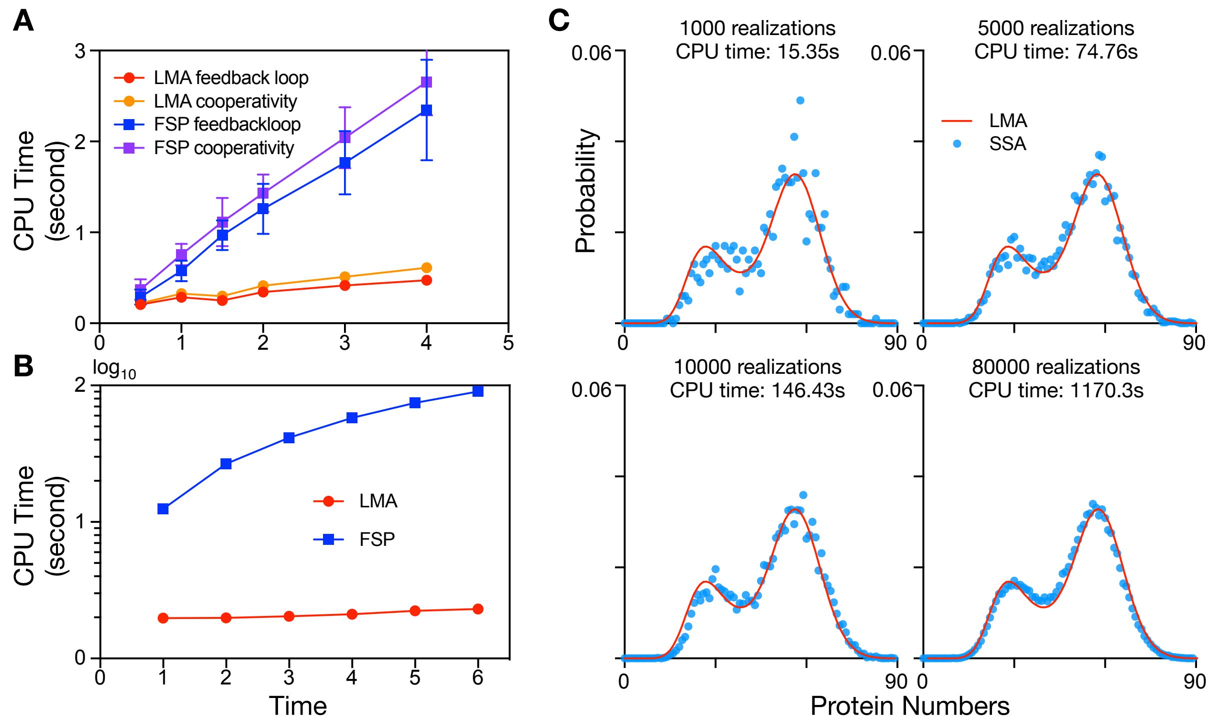

Supplementary Figure 2: Comparison of CPU time for the LMA, FSP and SSA methods. (A) The LMA and FSP are used to obtain the protein-distributions for the feedback loop with and without cooperativity for $t = 0.5, 1, 1.5, 2, 3, 4$ (these distributions are shown in Fig. 2D in the main text). The y-axis shows the corresponding CPU time for each algorithm for each value of t. The parameter set is $\rho_u = 60, \rho_b = 25, \sigma_u = 0.25, \sigma_b = 0.004$ and the FSP is solved in the protein number space $[0, 100]$ (this is the minimal truncation of state space which gives an accurate distribution). The error bars denote the standard deviation of CPU time conducted for 10 replicates. Note that while the LMA's CPU time is very weakly dependent on the time t at which protein distribution is evaluated, FSP's CPU time increases rapidly with increasing t. (B) Same as (A) but now using the parameter set $\rho_u = 240, \rho_b = 100, \sigma_u = 0.25, \sigma_b = 0.004$. This leads to protein distributions which are defined over the protein number space $[0, 300]$. The three times larger state space compared to (A) leads to a significant increase in the FSP's CPU time (from seconds to minutes) but the LMA's CPU time is barely affected and still of the order of few seconds. (C) The CPU time for the estimation of the protein distribution at time $t = 4$ using the SSA for the feedback loop for parameter set $\rho_u = 60, \rho_b = 25, \sigma_u = 0.25, \sigma_b = 0.004$. Note that to obtain an acceptable distribution a few thousand realizations are needed and the CPU time is of the order of minutes whereas the LMA's CPU time according to (A) for $t = 4$ is about 0.5 second.

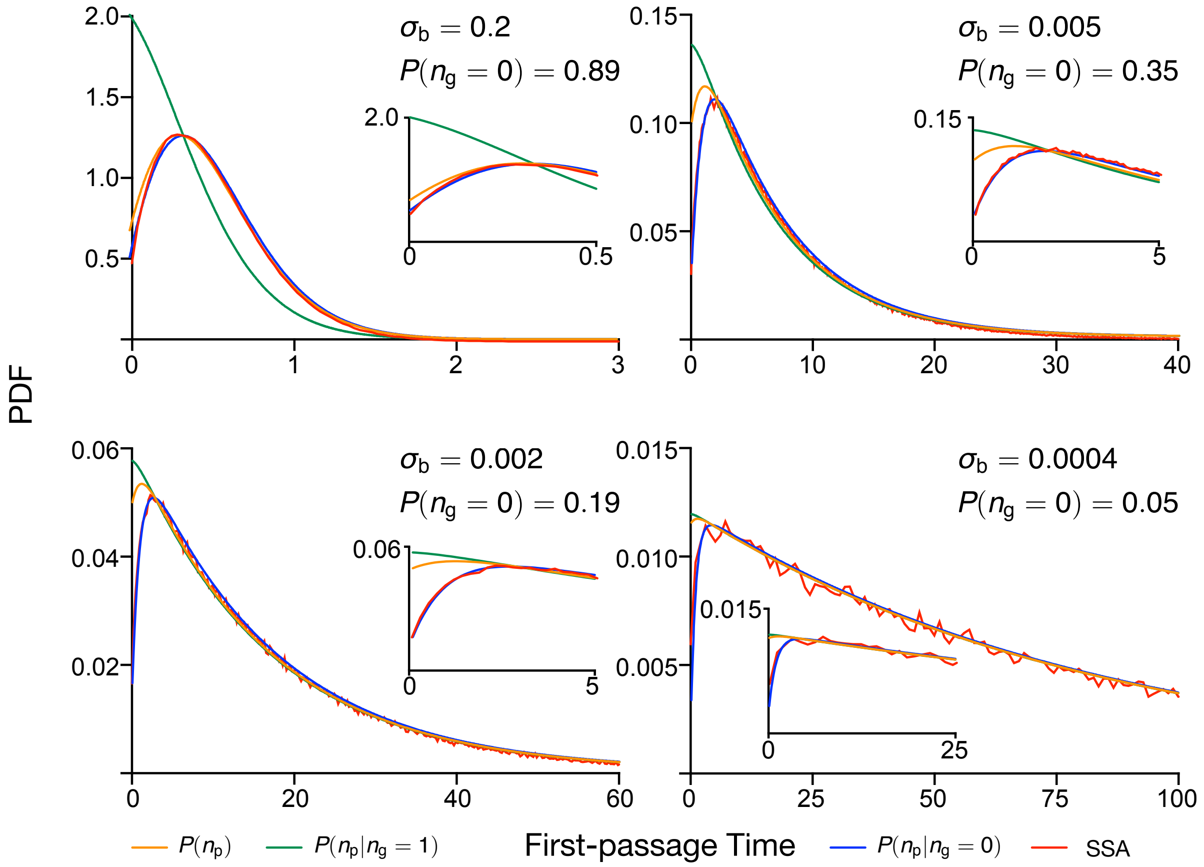

Supplementary Figure 3: First-passage time distribution for feedback loop without cooperativity for different values of σ_b. The distribution is calculated via Eq. (18), wherein Part A is calculated exactly and Part B uses one of three distinct steady-state probability distributions $P(n_p)$, $P(n_p|n_g = 1)$ or $P(n_p|n_g = 0)$. These probabilities are calculated exactly using the solution in [5]. Note that independent of the value of σ_b (and of $P(n_g)$, the probability of being in state G), the stochastic simulation algorithm (SSA) agrees exactly (within small sampling error) with Eq. (18) using $P(n_p|n_g = 0)$ for Part B.

Supplementary References

[1] Olver, F. W. J. *et. al.* Nist digital library of mathematical functions. http://dlmf.nist.gov/, Release 1.0.16 of 2017-09-18.

[2] Munsky, B. & Khammash, M. The finite state projection algorithm for the solution of the chemical master equation. J. Chem. Phys. **124**, 044104 (2006).

[3] Gillespie, D. T. Exact stochastic simulation of coupled chemical reactions. J. Phys. Chem. **81**, 2340–2361 (1977).

[4] Redner, S. *A guide to first-passage processes* (Cambridge Univ. Press, 2001).

[5] Grima, R., Schmidt, D. R. & Newman, T. J. Steady-state fluctuations of a genetic feedback loop: An exact solution. J. Chem. Phys. **137**, 035104 (2012).

[6] Gardner, T. S., Cantor, C. R., Collins, J. J. Construction of a genetic toggle switch in Escherichia Coli. *Nature* **403**, 339 (2000).

[7] Shahrezaei, V. & Swain, P. S. Analytical distributions for stochastic gene expression. Proc. Natl Acad. Sci. USA **105**, 17256–17261 (2008).

[8] Peccoud, J., Ycart, B. Markovian modeling of gene-product synthesis. *Theor. Popul. Biol.* **48**, 222–234 (1995).

[9] Raj, A. *et al.* Stochastic mRNA synthesis in mammalian cells. *PLoS Biol.* **4**, e309 (2006).

[10] Khalil, H. K. Nonlinear Systems (3rd edition, Prentice-Hall, 2002).
